# Open-Access Metabolomics Databases for Natural Product Research: Present Capabilities and Future Potential

**DOI:** 10.3389/fbioe.2015.00022

**Published:** 2015-03-04

**Authors:** Sean R. Johnson, Bernd Markus Lange

**Affiliations:** ^1^Institute of Biological Chemistry, M.J. Murdock Metabolomics Laboratory, Washington State University, Pullman, WA, USA

**Keywords:** gas chromatography, high performance liquid chromatography, mass spectrometry, metabolomics, natural product, nuclear magnetic resonance spectroscopy, secondary metabolite

## Abstract

Various databases have been developed to aid in assigning structures to spectral peaks observed in metabolomics experiments. In this review article, we discuss the utility of currently available open-access spectral and chemical databases for natural products discovery. We also provide recommendations on how the research community can contribute to further improvements.

## Introduction

In the broadest sense, a natural product is a chemical compound (metabolite) synthesized by a living organism. However, for the current review article, we will use the more restrictive definition of a metabolite involved in secondary or specialized metabolism (as opposed to primary metabolites of central metabolism in all organisms). Tens of thousands of structurally diverse natural products have been isolated and tested for drug discovery, many of them with unique mechanisms of action (Harvey, 2008). Medicinal chemists employ natural products as structural scaffolds for the synthesis of analogs in the development of drugs with improved pharmacological potency and safety (Cragg and Newman, 2013). In the environment, natural products are of paramount importance for the interactions between different organisms [e.g., defense antibiotics in microbes; Duffy et al. (2003)] and communication among members of a species [e.g., sex pheromones in insects; Leal (2013)].

**Figure 1:**
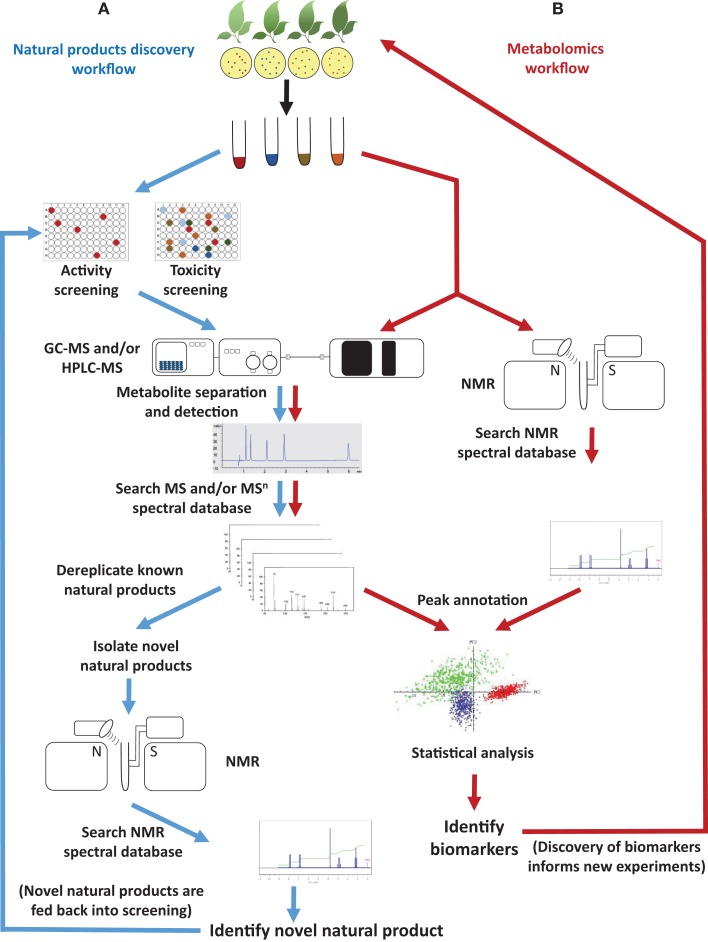
**Typical workflow of (A) natural products discovery and (B) metabolomics projects**. Common objectives include metabolite identification by searching spectral databases.

**Figure 2:**
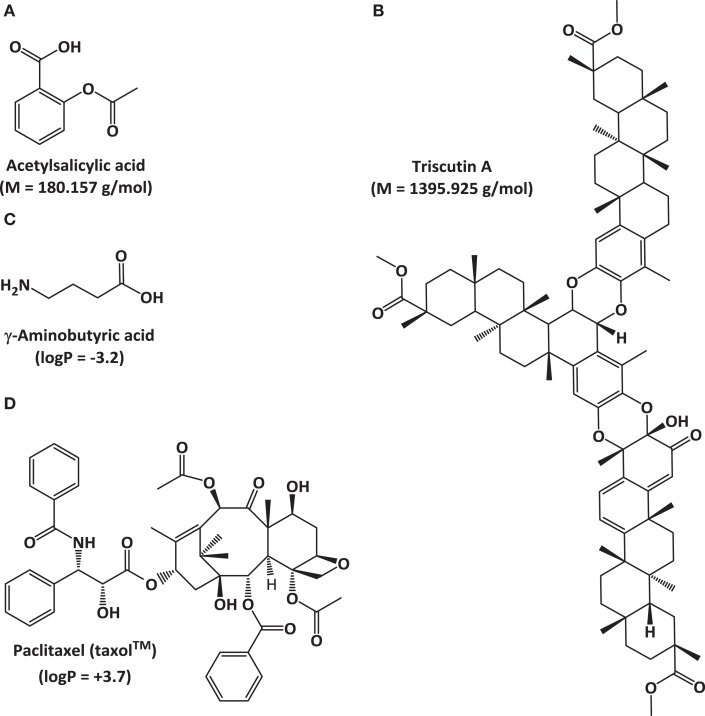
**Examples of natural products with vast different physicochemical properties to illustrate challenges for analytical chemistry**. Molecular weight differences: **(A)** acetyl salicylic acid and **(B)** triscutin A. Polarity differences: **(C)** γ-aminobutyric acid and **(D)** paclitaxel (taxol^®^).

The fields of natural products discovery and metabolomics evolved independently and emphasize different aspects of metabolite analysis: while the former focuses on identifying individual, bioactive metabolites (e.g., novel drug candidates), the latter seeks to extract meaning from extraordinarily complex data sets (e.g., biomarkers indicative of a particular biological state) (Figure 1). Despite a historic division, both fields have partially overlapping objectives (e.g., metabolite identification) and rely on the same analytical technologies (Robinette et al., 2012). The most commonly employed approaches involve mass spectrometry (MS), often in conjunction with prior chromatographic separation, and nuclear magnetic resonance (NMR) (Figure 1). Despite considerable technological advances in metabolite separation and analysis, the immense array of natural product structures and chemical properties presents formidable challenges to analytical chemists. For example, some bioactive metabolites are quite small [e.g., acetylsalicylic acid (aspirin^®^; polyphenol with anti-inflammatory properties) at 180.157 g/mol], while others are rather large molecules [e.g., triscutin A (triterpenoid trimer occurring in the Celastraceae) at 1,395.925 g/mol]. Some are highly hydrophilic [e.g., γ-aminobutyric acid (GABA; neurotransmitter) with a logP of -3.2], while others are lipophilic [e.g., paclitaxel (taxol^®^); anticancer diterpene with a logP of +3.7] (Figure 2).

The convergence of metabolomics and natural product discovery occurs at the stage when spectral databases are searched with physicochemical parameters (e.g., relative retention time, mass-over-charge ratio, mass spectral fragmentation patterns, and/or NMR spectral peaks) determined for complex mixtures or isolated metabolites (Figure 1). The quality of peak annotation (assigning a chemical identity or unique identifier) in metabolomics experiments and dereplication (recognizing and eliminating from further consideration metabolites with known structures) in natural product screening are critically dependent on the completeness and accessibility of spectral databases. For decades, natural products researchers have relied mostly on commercial databases (e.g., NIST Standard Reference MS Database and Aldrich Spectral Viewer^®^ NMR Library) or have developed custom, in-house, databases with limited or no public access. The metabolomics field, in contrast, has taken a radically different approach and has embraced open-access databases and data exchange for all aspects of the experimental process, from sample tracking, to data analysis algorithms, and finally to meta-data deposition. Access-restricted commercial libraries will likely continue to be an important tool in natural products research in the future but, because content and search algorithms are proprietary, they are very difficult to evaluate. The focus of the present review is therefore to discuss the utility of currently available open-access spectral databases (accessed between August and November 2014) in the context of natural product identification and to suggest steps toward a better integration of natural products discovery and metabolomics.

## Mass Spectrometry-Based Spectral Databases

In natural products discovery, metabolite separation [mostly using gas chromatography (GC) or high performance liquid chromatography (HPLC)] is generally combined with detection by on-line MS (usually termed GC-MS or HPLC-MS, respectively). Fragmentation patterns in MS are reflective of structural properties, and it is thus common to record multiple-stage MS data with two (MS/MS or MS^2^) or more product spectra (MS*^n^*, where *n* is the number of product ion stages) (Murray et al., 2013). Recent developments have resulted in improved chromatographic resolution (narrower peaks and shorter chromatographic runs) and enhanced MS detection (higher sensitivity, acquisition speed, mass resolution, and mass accuracy). Because of these desirable properties, GC-MS and HPLC-MS are also workhorse technologies for metabolomics efforts (Zhang et al., 2012). Several recent reviews have discussed the general capabilities of various MS-based databases and software tools (Tohge and Fernie, 2009; Kind and Fiehn, 2010; Sugimoto et al., 2012; Scheubert et al., 2013). The focus of this section is therefore an evaluation of the utility of these online resources for natural products discovery (rather than profiling cellular metabolism or differentiating patterns).

The Golm Metabolome Database (GMD; Kopka et al., 2005; Schauer et al., 2005) is a GC-MS database combining GC retention indices (retention times relative to a set of standards) and electron impact (EI) mass spectra, all acquired under defined conditions, as mass spectral tags (MSTs) for both neat authentic standards as well as plant extracts (Table 1). When users search their own plant extract data against the GMD database, MSTs in their query can be matched to records of either known metabolites or unique identifiers for unidentified peaks in complex plant extracts. The inclusion of unidentified MSTs in the database allows peaks to be compared across samples, even when the identity of the metabolites generating these peaks is unknown. Queries can be submitted to GMD through a web form, or automated through a scripting application program interface (API). Another useful feature of GMD is the decision tree search (Hummel et al., 2010), which predicts substructures likely to be present in an unknown metabolite, even if the complete structure cannot be determined.

**Table 1:** **Online spectral databases for natural product identification**.

Database	URL	Search parameters	Compounds	Spectra	Record format	Accepts submissions	Notes	Reference
BML-NMR	http://www.bml-nmr.org/	Name	208	3,328 NMR	Vendor specific, MSI-XML	No	Each compound measured with 16 different NMR parameter sets	Ludwig et al. (2012)
BMRB	http://www.bmrb.wisc.edu/metabolomics/	Name, mass, structure, ^13^C-NMR shifts, ^1^H-NMR shifts, HSQC-NMR peaks, other	1,249	8,996 NMR	NMR-STAR, CSV, vendor specific	Yes		Ulrich et al. (2008)
GMD	http://gmd.mpimp-golm.mpg.de	Name, mass, formula, functional group, MS peaks, retention index, other	2,220	26,587 MS	NIST, JCAMP-DX, TagFinder, Target Search	Yes	GC retention indexes. Multiparameter search interface. Decision tree tool for substructure identification. API access	Wagner et al. (2003); Kopka et al. (2005)
GNPS	http://gnps.ucsd.edu	MS^2^ in mzML format, name, adduct, other	>5,500	27,593 MS^2^	mgf	Yes	Automated dereplication workflow from MS^2^ data	Unpublished
HMDB	http://www.hmdb.ca/	Structure, mass, adduct, MS peaks, MS^2^ peaks, GC retention time, GC retention index, ^1^H-NMR shifts, ^13^C-NMR shifts, 2D TOCSY ^13^C HSQC other	41,806	2,240 NMR; 1,220 MS; 8,176 MS^2^	Text, vendorspecific, NIST	No	Specific for human metabolites. Not all compounds have experimental spectra	Wishart et al. (2013)
MassBank	http://www.massbank.jp/	Structure, name, mass, formula, fragment, MS(n) peaks, neutral loss	>11,000	40,889 MS including MSn	MassBank	Yes	Many of the records also include detailed information about chromatographic conditions and retention times. Batch search available. SOAP API	Horai et al. (2010)
METLIN	http://metlin.scripps.edu	Mass, adduct, fragment, name, formula, neutral loss, MS^2^ peaks	240,515	61,872 MS^2^	Not downloadable	No	Batch search available. Not all compounds have experimental spectra	Smith et al. (2005)
MMCD	http://mmcd.nmrfam.wisc.edu/	Name, structure, NMR shifts and connectivity, mass, adduct	20,306	5,256 NMR	Text, vendor specific	No	Batch search available. Can use multiple kinds of spectra in a single search. Not all compounds have experimental spectra	Cui et al. (2008)
NAPROC-13	http://c13.usal.es/c13	Name, chemical family, formula, mass, publication, ^13^C shift, and multiplicity	20,297	20,297 NMR	Not downloadable	No	Iterative search where shifts can be added to the search one at a time. Search by shift connectivity	López-Pérez et al. (2007)
NMR ShiftDB	http://nmrshiftdb.nmr.uni-koeln.de/	Name, formula, citation, structure, NMR shifts (multiple nuclei), experimental conditions	42,838	50,883 NMR	CML, JCAMP-DX, tab separated, SQL	Yes	Lists NMR chemical shifts, but not peak size. Database is available from the SourceForge page	Steinbeck et al. (2003); Steinbeck and Kuhn (2004)
ReSpect	http://spectra.psc.riken.jp/	Mass, adduct, fragment, name, keyword, formula, MS(n) spectrum	3,710	9,017 MSn spectra	MassBank	No	Specific for phytochemicals	Sawada et al. (2012)
SDBS	http://sdbs.db.aist.go.jp	Name, formula, mass, IR peaks, ^13^C-NMR shifts, ^1^H-NMR shifts, MS peaks	34,000	29,000 NMR; 24,700 MS	Not downloadable	No	Can use multiple kinds of spectra in a single search. Limit of 50 searches per day	Yamamoto et al. (1988)
Spektraris	http://langelabtools.wsu.edu/spektraris/	Mass, retention time, relative retention time, MS(n) peaks, formula, ^1^H-NMR shifts, ^13^C-NMR shifts	733	466 NMR; 1,445 MS; 1,181 MS^2^	MassBank, tab separated, JCAMP-DX, vendor specific	Yes	Multiple parameter search interface. NMR data currently limited to taxane diterpenes	Cuthbertson et al. (2013); Fischedick et al. (2015)
SpinAssign	http://prime.psc.riken.jp/	^13^C-HSQC NMR shifts, ^1^H-NMR shifts, ^13^C-NMR shifts	980	980 NMR	Not downloadable	No	Optimized for mixtures	Chikayama et al. (2010)

*“Compounds” count all compounds that could be returned as a search hit, including those for which no experimental spectra are available. “Spectra” counts only experimentally measured spectra. Databases surveyed in September and October 2014*.

METLIN is a comprehensive MS/MS (or MS^2^) database that contains about 62,000 spectra representing more than 12,000 metabolites (plus a large number of theoretical spectra) (Table 1). All spectra were acquired under standardized conditions (electrospray ionization, positive and negative polarity, high mass accuracy, four different collision energies) on a quadrupole time-of-flight (QTOF) mass spectrometer (Smith et al., 2005; Benton et al., 2008). The database includes predicted masses and elemental formulas for a total of over 240,000 entries (the vast majority of which does not appear to be natural products), and batch queries with multiple spectra are possible. The lack of chromatographic data and closed design (no records or data can be downloaded) are limitations.

MassBank is a comprehensive database with access to data acquired with various (mostly high resolution) MS-based platforms (GC-MS and HPLC-MS) (Horai et al., 2010). The database records can be searched through an online interface or with the Mass++ software (Tanaka et al., 2014). MassBank also has a simple object access portal (SOAP) interface, allowing queries to be submitted programmatically. All MassBank records have spectral information, and some also include chromatographic information; however, retention times (or indices) cannot be used as a search parameter in the current MassBank online interface.

ReSpect (Sawada et al., 2012) is an MS^2^ database specific to plant metabolites (Table 1). For natural products discovery, a particular advantage of ReSpect is that spectral records are annotated with taxonomic information about the species from which a particular metabolite has been extracted and to which structural class the metabolite belongs.

The Global Natural Products Social Molecular Networking resource (GNPS^1^; unpublished) is an MS^2^ database with an emphasis on natural products of all biological origins (Table 1). In addition to unique spectral records, some spectra from MassBank and ReSpect are also included. The GNPS website features a computational tool to facilitate the dereplication process.

MetFrag is an online tool that performs *in silico* fragmentations, allowing searches of experimental MS spectra against chemical databases such as KEGG, PubChem, ChemSpider, or custom structure databases uploaded in .sdf format (Wolf et al., 2010).

Other databases with MS data are HMDB (Wishart et al., 2013), MMCD (Cui et al., 2008), SDBS^2^, and Spektraris (Cuthbertson et al., 2013; Fischedick et al., 2015), which are discussed in more detail below (see Section “Multi-Parameter Databases”).

## Nuclear Magnetic Resonance-Based Spectral Databases

Nuclear magnetic resonance (NMR) is the gold standard for compound structure elucidation, but is inherently slower and less sensitive than MS, and on-line HPLC-NMR is therefore not a routine technology. In a typical NMR workflow for natural product discovery, fractions with metabolites of interest are collected from chromatographic runs and then processed for off-line NMR analysis. Metabolomics applications are generally focused on the NMR-based analysis of extracts or body fluids without prior chromatographic separation. NMR databases and computational methods in metabolite identification have been reviewed by Ellinger et al. (2012) and Halabalaki et al. (2014). Here we discuss which open-access databases are particularly useful for natural products discovery.

For the purpose of dereplication, NAPROC-13 is noteworthy because of its large collection of natural product spectra (>20,000) and inclusion of metabolite classification (López-Pérez et al., 2007). Significant limitations are the closed design (no user access to spectral data or even a list of compounds) and the fact that ^13^C-NMR spectra and other parameters (e.g., molecular formula or predicted molecular weight) can only be searched separately (but not as orthogonal parameters). NMRShiftDB (Steinbeck et al., 2003; Steinbeck and Kuhn, 2004) and SDBS (Yamamoto et al., 1988) are not limited to natural products, but are notable because of the size of their spectral collections, with approximately 51,000 and 29,000 spectra respectively. The BML-NMR database covers only 203 compounds but the spectral depth (16 different one- and two-dimensional experiments for each compound) provides high quality references (Ludwig et al., 2012). BMRB contains NMR data for various biomolecules with a focus on protein, peptide, and nucleic acid spectra (Ulrich et al., 2008). However, over the last few years, more than 1,200 spectra obtained with metabolites (mostly from plant metabolism) have been added. The database is particularly rich in metabolites involved in the biosynthesis of lignin (plant cell wall constituent) and those obtained by plant cell wall deconstruction. Spectra are available for downloading in raw and processed data formats. More discussion of other databases housing NMR data [e.g., HMDB (Wishart et al., 2013), MMCD (Cui et al., 2008), SDBS (see text footnote 2), and Spektraris (Cuthbertson et al., 2013; Fischedick et al., 2015)] is provided below (see Section “Multi-Parameter Databases”).

To search against any of the above-mentioned databases, NMR spectra must be processed and interpreted using external software, and entered into a web interface as a peak table (direct spectral comparisons are currently only offered in proprietary packages that do not follow the open-access model and are thus not reviewed here). The rNMR software (Lewis et al., 2009) interfaces with the MMCD database by allowing the export of spectral peaks to a searchable file format. Searches of online databases generally work best when the query spectrum is from a neat standard. In contrast, SpinAssign is designed to identify individual compounds from ^13^C-HSQC spectra of complex cell extracts (Chikayama et al., 2010). The MetaboHunter and COLMAR tools can be used to search NMR spectra of mixtures against those of a subset of metabolites in HMDB and BMRB (Robinette et al., 2008; Tulpan et al., 2011; Bingol et al., 2012, 2014). CSEARCH enables searches against computationally predicted ^13^C-NMR chemical shift values for compounds represented in large public databases such as PubChem (Kalchhauser and Robien, 1985). This tool is convenient for categorizing metabolites by class, but further spectral interpretation or data gathering is required to afford unequivocal structure assignments.

## Multi-Parameter Databases

Comparisons of multiple types of chromatographic and/or spectral parameters (e.g., MS^2^ spectral patterns and NMR shift values in one search) are an effective means to lower the risk of false positive identifications and increase the confidence in dereplication results (Blunt and Munro, 2013). According to recommendations by the Metabolomics Standards Initiative (MSI) (Sumner et al., 2007), the identification of a compound at the highest confidence level (MSI level 1) requires comparisons of orthogonal parameters of the unknown to an authentic standard measured in the same laboratory and under the same conditions. A database search alone permits, at best, an identification at MSI level 2 (“putatively annotated compound”). The distinction between MSI level 2 and MSI level 3 (“putatively characterized compound classes”) is to some extent a judgment call on the part of the investigator, as both annotations are based on comparisons to the chemical literature and spectral databases. The goal of dereplication efforts in natural products discovery is to eliminate from further consideration, those compounds that have been previously identified. In other words, a primary interest of the natural products chemist is to distinguish compounds that can be given an MSI level 2 identification from those that are annotated with MSI levels 3 or 4 (“unknown compounds”), so that further efforts can be focused on structural elucidation of compounds likely to be novel. In this section we discuss databases that allow simultaneous searches with multiple orthogonal parameters.

Several databases with very large numbers of spectral records, for example NMRShiftDB (Steinbeck et al., 2003; Steinbeck and Kuhn, 2004) and SDBS (Yamamoto et al., 1988), allow searches with multiple spectrum types in a single query. However, because these databases contain a relatively small number of natural products spectra (compared to those of synthetic compounds), the record lists returned from spectral searches often contain larger numbers of compounds that do not occur in nature. HMDB has highly informative records with excellent spectral data and offers a query interface that can use complex Boolean combinations to search record parameters (Wishart et al., 2013). MMCD contains experimental NMR data (one- and two-dimensional) for approximately 800 metabolites (Cui et al., 2008). An additional 1,200 NMR data sets were taken from the literature and 300 spectra predicted computationally. The web interface allows searches with multiple parameters (NMR, exact mass and/or molecular formula). Both MMCD and HMDB include a web-based tool that allows MS spectral comparisons against predicted spectra (accurate mass and adducts commonly encountered in HPLC-MS), which can be helpful in dereplication efforts with extracts whose constituents have not yet been incorporated into databases. The Spektraris tool contains HPLC-MS [combination of retention time relative to an internal standard and mass-over-charge ratio (accurate mass-time tag)] and NMR (^1^H and ^13^C) data (Cuthbertson et al., 2013; Fischedick et al., 2015). MS^2^ data were also acquired under the same conditions, and both MS and MS^2^ spectra were submitted to MassBank. Spektraris is focused on natural products (more than 700 metabolites), and therefore has the potential to be useful for natural products dereplication efforts. The Spektraris-NMR database contains very well annotated records but is currently limited to taxanes, a larger class of natural products found in the Taxaceae (yew family).

## Metabolite Databases without Spectral Data

The distribution of natural products is often limited to certain taxa. In the section on MS-based databases, we mentioned ReSpect as a positive example for the incorporation of biological source information. However, knowledge regarding the occurrence of natural products across taxa, although potentially useful for dereplication, is generally difficult to find in a single location.

The KNApSAcK database aims to change this and has launched an ambitious effort to catalog the association of metabolites with taxonomic information (Afendi et al., 2012). The output is a list of metabolite-species pairs. MS spectra can be searched against theoretical metabolite masses and mass-over-charge ratios of possible MS adducts (predicted based on metabolite structure). However, while searchable online, the database records cannot be downloaded.

The Universal Natural Products Database (UNPD) provides access to chemical information relevant for virtual activity screening of a large number (>200,000) of natural products (Gu et al., 2013). Hits from metabolite searches can be linked to taxonomic information online but only chemical records are downloadable. Other databases that enable metabolite-to-species correlations are KEGG (Kanehisa et al., 2014) and MetaCyc (Caspi et al., 2014). Both databases focus on metabolic pathways and only metabolites with associated reactions are included. As a note of caution, these databases infer the occurrence of metabolites in certain species from computationally annotated genome or transcriptome sequences, which may or may not have been confirmed chemically.

SuperNatural is a comprehensive natural products database (>300,000 metabolites) with information about chemical structures, structural relatedness, mechanism of action, metabolite-target pairs, and commercial availability (Dunkel, 2006; Banerjee et al., 2015).

The ZINC database is a repository generated from records of various databases and can be searched by selecting “Natural Products” as a subset (Irwin et al., 2012). ZINC also lists commercial availability.

PubChem is one of the most versatile and comprehensive databases for chemical compounds (Bolton et al., 2008). However, searches with a molecular weight or elemental formula of a natural product often return long lists of records of chemically synthesized compounds that do not occur in nature.

ChEBI is a manually curated database of chemicals of biological interest (Hastings et al., 2013). As a consequence of these curation efforts, it contains fewer metabolite records than some other structural databases, but the annotations for each compound are very rich.

Some databases were developed to capture structural diversity in certain geographical areas. For example, AfroDB provides downloadable structural records for more than 1,000 natural products extracted from African plants (Ntie-Kang et al., 2013), but there is no online search interface. The TCM Database@Taiwan contains structural records for more than 20,000 metabolites isolated from traditional Chinese medicines (Chen, 2011). Chemical data is available for download, but not for taxonomic information.

As algorithms for the computational prediction of mass and NMR spectra from structures improve, the utility of chemical structure databases lacking experimental spectra will likely increase. However, it will be important to provide more information about the provenance of data, in particular which organisms the compounds were isolated from, where to find literature references to the primary data, and which methods were used in structural identification.

## Coverage of Open-Access Spectral Databases

To evaluate the coverage and uniqueness of presently available natural products spectral databases, we assessed those that allow bulk downloads of spectral records (Table 2). NMRShiftDB was excluded from the analysis because of the very large number of synthetic compounds and comparatively low number of natural products, and low overlap with all other databases. Because data formats in natural product databases are not standardized, we used custom Python scripts to extract structural information, which was then converted into IUPAC International Chemical Identifier (InChI) format using Molconvert (ChemAxon, Budapest, Hungary). Database records with only a metabolite name and/or CAS number were left out due to the difficulty in converting these to InChI format. In addition, stereochemical information was not always available, and we therefore based our definition of a unique structure only on the “Main” layer of the InChI, which includes the formula and atom connectivity. For these reasons, the compound numbers reported here can be much lower than those reported on the project websites or in publications.

**Table 2:** **Overlap of coverage (in percent) among open-access chemical databases with a bulk download option (one-versus-one comparison)**.

Database	Total number of compounds	BML-NMR	BMRB	GMD	GNPS	HMDB	MassBank	ReSpect	Spektraris
BML-NMR	199		79	60	85	88	90	79	14
BMRB	1,159	14		29	35	39	52	27	7
GMD	879	14	38		48	62	66	35	10
GNPS	5,105	3	8	8		11	40	14	7
HMDB	1,046	17	43	52	54		68	38	9
MassBank	11,012	2	6	5	19	6		6	4
ReSpect	718	22	43	43	100	56	89		13
Spektraris	723	4	11	12	52	13	67	12	
Combined (unique)	15,247								

*“Total” refers to the number of unique chemicals annotated with a structure and associated with at least one MS or NMR spectrum. Example: BMRB contains spectra for 1,159 structure-annotated unique chemical entities. Of these, 29% are also found in GMD. GMD contains spectra for 879 structure-annotated unique chemical entities. Of these, 38% are also found in BMRB*.

The number of unique chemical structures in the combined database records is approximately 15,000 (Table 2). As expected, databases that emphasize natural products, such as GNPS, MassBank, and Spektraris have relatively low overlap with other databases. The high degree of overlap among these three databases is due to the fact that all MS and MS^2^ spectra from Spektraris were also submitted to MassBank, and GNPS subsequently incorporated many of the publicly available MassBank spectra, including some that originated from Spektraris. The relatively high degree of overlap among BMRB, GMD, HMDB, and ReSpect is likely due to their mutual coverage of primary metabolites (which occur in all organisms). Mass spectra and tandem mass spectra are by far the most readily available data sets for natural products (approximately 9,600 and 6,300, respectively) (Table 3). ^1^H-, ^13^C-, and two-dimensional NMR spectral records are at hand for a much smaller number of metabolites (approximately 1,800, 1,400, and 1,200, respectively), which is likely due to the fact that larger amounts (microgram to milligram range) of neat standards are required to obtain high quality NMR spectra within a reasonable time frame.

**Table 3:** **Spectral data available in open-access chemical databases with a bulk download option**.

Database	MS	MS*^n^*	^1^H-NMR	^13^C-NMR	2D-NMR
BML-NMR	0	0	199	0	199
BMRB	0	0	1,153	1, 154	755
GMD	879	0	0	0	0
GNPS	0	5,105	0	0	0
HMDB	255	971	823	109	815
MassBank	9,241	2,736	0	0	0
ReSpect	0	718	0	0	0
Spektraris	482	311	240	216	0
Combined (unique)	9,651	6,333	1,829	1, 383	1, 183

*The number of unique chemicals annotated with a structure and associated with at least one MS or NMR spectrum is given*.

A feature of great interest for natural products researchers is the diversity of compound classes represented in a spectral database. Unfortunately, this information is generally not reported in a standardized format (or not at all); so, a robust and systematic evaluation of database contents is not feasible. However, based on the available data, a few general statements can be made. The emphasis of BML-NMR is on human metabolites, with very limited coverage of natural products (Table 4). BMRB focuses on plant metabolites, with strength in the coverage of metabolites related to the formation of cell walls. HMDB contains mostly human metabolites (and only a few dietary natural products), with lipids and amino acid derivatives featuring prominently (Table 4). NAPROC-13 incorporates spectra for compounds representing all major natural product classes (>15,000 according to the developers), and would seem to stand out among open-access spectral databases both in terms of breadth and depth (Table 4). However, this information cannot be confirmed independently because, although search functions are provided without restrictions, there is no access to NAPROC-13 data or compound lists. ReSpect contains information about close to 4,000 plant natural products with an emphasis on flavonoids (>1,300 metabolites), terpenoids (>500 metabolites), phenylpropanoids (>300 metabolites), and alkaloids (>250 metabolites). Spektraris-AMT provides access to mass spectral data of members of the major plant natural product classes, while Spektraris-NMR currently contains NMR spectral data and detailed annotation for only one natural product class (taxane diterpenes) (Table 4). In summary, significant progress has been made with populating online databases with spectral records for natural products but, to enhance their overall utility for the research community, efforts to provide complete annotations and open access to the data will need to continue.

**Table 4:** **Major compound classes represented in open-access spectral databases**.

Database	Major compound classes	Data source
BML-NMR	Focus on human metabolites	Discussion in Ludwig et al. (2012) and a manual inspection of compound list
BMRB	Plant cell wall components (486) plus various other plant metabolites	Information on project website
HMDB	Focus on human metabolites. Of the 1,046 compounds associated with spectral data, 253 are lipids (e.g., fatty acids, steroids, and prenol lipids) and 174 are amino acid derivatives	Metabolite class annotation available
NAPROC-13	Terpenoids are the best represented compound class (15,527). Other well-represented classes are steroids (729), flavonoids (1,769), “aromatics” (1,236), chromans (304), and lignans (294)	Metabolite class annotation available
ReSpect	Focus on plant metabolites. Flavonoids are the best represented class (1,360). Other well-represented classes are terpenoids (519), phenylpropanoids (341), alkaloids (256), amino acid derivatives (236), and glucosinolates (93)	Metabolite class annotation available
Spektraris	Focus on plant metabolites. MS spectra for alkaloids (>100), flavonoids (>80), lignans and phenylpropanoids (>60), and terpenoids (>50). NMR spectra for terpenoids (248)	All of the NMR spectra are for taxanes. The MS spectra are not annotated with compound class

## Critical Need for Spectral Record Standardization and a Centralized Data Repository

New ways of searching and using chemical data are constantly being developed. When the open-access model is employed for data dissemination, innovative tools with novel functionalities can be developed. For example, the search interfaces provided for the HMDB and BMRB websites are not optimized for identifying individual metabolites in complex extracts. The MetaboHunter and COLMAR family of tools use the same spectral records but with algorithms to extract information from data acquired with mixtures. Another example is GNPS, which combines its own spectra with those from MassBank and ReSpect for use in an MS^2^-based dereplication workflow.

The need for the adoption of a standardized record format, adherence to community standards for data dissemination, and creation of a central repository for metabolomics data has been reiterated by various authors (Kind et al., 2009; Griffin and Steinbeck, 2010; Kim et al., 2011; Goeddel and Patti, 2012; Nicholls, 2012). Here we would like to emphasize that it is particularly important to provide unambiguous chemical structure identifiers in SMILES (Anderson et al., 1987), Molfile, and InChI (Heller et al., 2013) formats. However, even if future studies were to adhere to these standards, there is still the issue of incorporating older data sets, particularly because opportunities to re-acquire spectra for exotic, low abundance, natural products are limited. NAPROC-13, ReSpect, and Spektraris-NMR records were generated by the slow process of transcribing spectra from figures and peak listings found in the literature; a process that, albeit cumbersome, will need to continue. While a standard central repository for metabolite standards is still lacking, a number of existing databases accept external submissions (Table 1) and can be used by researchers wanting to make their data available without having to develop their own infrastructure.

## Conclusion

Open-access spectral databases are potentially very useful to natural products researchers. However, the coverage of spectra in these databases is small compared to the total number of known natural products. We have pointed out ways in which existing databases can be employed for natural products dereplication and have suggested steps that natural products scientists can take to contribute to spectral cataloging efforts. An open-access and standards-based approach to data acquisition and reporting will allow data exchange between different resources and the development of enhanced tools by the community, thereby improving accuracy, coverage, and functionality. Professional organizations and publishers should support the deposition of standardized records to centralized repositories, akin to the submission of sequences to GenBank or EMBL. To ensure broad participation by researchers, the entire process of data deposition, from converting vendor-specific raw data files to generating standardized spectral records, will have to be simplified. An inclusive, community-based approach to the further development of spectral resources has the potential to speed up natural products discovery significantly.

## Conflict of Interest Statement

The authors declare that the research was conducted in the absence of any commercial or financial relationships that could be construed as a potential conflict of interest.

## Footnotes

^1^http://gnps.ucsd.edu

^2^http://sdbs.db.aist.go.jp
